# Analysis of the Relationship Between Glycated Hemoglobin and Echocardiographic Parameters in Patients Without Diabetes: A Retrospective Study

**DOI:** 10.3390/jcm15010033

**Published:** 2025-12-20

**Authors:** Grzegorz K. Jakubiak, Natalia Pawlas, Monika Starzak, Dominika Blachut, Artur Chwalba, Celina Wojciechowska, Grzegorz Cieślar

**Affiliations:** 1 Department of Pharmacology, Faculty of Medical Sciences in Zabrze, Medical University of Silesia in Katowice, Jordana 38 St., 41-808 Zabrze, Poland; n-pawlas@wp.pl (N.P.); artur.adam.chwalba@gmail.com (A.C.); 2 Department of Internal Medicine, Angiology and Physical Medicine, Faculty of Medical Sciences in Zabrze, Medical University of Silesia in Katowice, Batorego 15 St., 41-902 Bytom, Poland; mslekarz@gmail.com (M.S.); cieslar1@tlen.pl (G.C.); 3 2nd Department of Cardiology, Faculty of Medical Sciences in Zabrze, Medical University of Silesia in Katowice, Skłodowskiej-Curie 10 St., 41-800 Zabrze, Poland; dominikadyrcz@gmail.com (D.B.); cwojciechowska@sum.edu.pl (C.W.)

**Keywords:** glycated hemoglobin, non-diabetic patients, echocardiography, cardiovascular disease, subclinical cardiovascular dysfunction

## Abstract

**Background:** Glycated hemoglobin (HbA1c) is a parameter commonly used in clinical practice to assess glycemic control in patients with diagnosed diabetes. Hyperglycemia is a strong risk factor for developing cardiovascular (CV) disease. Although there is some evidence that this parameter could also help assess CV health in patients without known carbohydrate metabolism disorders, this is not entirely clear. The purpose of this study was to investigate the relationship between HbA1c and selected echocardiographic parameters in patients without diabetes. **Methods:** This study was a retrospective analysis of data from 59 patients (females: 72.88%) with a mean age of 54.82 ± 17.34 years without any features of acute illness or exacerbation of chronic diseases hospitalized in the Department of Internal Medicine, Angiology and Physical Medicine of the Medical University of Silesia in Katowice (Poland) in the period between June 2022 and May 2024. Only individuals with HbA1c levels and who have undergone transthoracic echocardiography were included in the analysis. Spearman’s rank correlation test was used for statistical analysis, and a multivariate analysis model was then constructed (adjusted for age, sex, body mass index, low-density lipoprotein cholesterol, systolic blood pressure, hypertension, and smoking). **Results:** In univariate analysis, HbA1c was found to be significantly correlated with selected parameters relating to left ventricular dimensions and mass, left atrial dimensions, right ventricular systolic function, mitral inflow profile parameters, and tissue Doppler echocardiography. Multivariate analysis did not confirm a significant association between HbA1c and the assessed echocardiographic parameters. **Conclusions**: Although HbA1c significantly correlates with some echocardiographic parameters, the observed relationships are entirely explained by confounding variables.

## 1. Introduction

Cardiovascular diseases (CVDs), especially those developing in the course of atherosclerosis, are among the leading causes of morbidity and mortality worldwide [1]. Deepening understanding of CVD pathogenesis is essential and may lead to more effective prevention of cardiovascular (CV) events. In the context of the growing epidemic of obesity and cardiometabolic disorders, studies on mechanisms linking metabolic disturbances with the cardiac phenotype are of particular importance.

With increasing age, the heart undergoes complex structural and functional changes referred to as cardiac aging. These include diastolic dysfunction, impaired relaxation and ventricular filling, and alterations in left-atrial function, all of which increase CV risk [2,3]. Transthoracic echocardiography (TTE) remains a fundamental diagnostic tool that enables precise assessment of cardiac chamber morphology and valve function [4].

Diabetes is one of the most important modifiable CV risk factors [5]. The accompanying hyperglycemia promotes fibrosis, adverse remodeling, and impaired myocardial mechanics [6]. Glycated hemoglobin (HbA1c) remains the primary marker of long-term glycemic control in clinical practice, because it reflects average blood glucose over the preceding three months; it is simple, inexpensive, and widely available [7,8]. HbA1c levels increase with age regardless of glycemic status [9,10]. Mechanistically, chronic hyperglycemia and glycation intensify oxidative stress, inflammation, and the accumulation of advanced glycation end products (AGEs), thereby increasing vascular and myocardial stiffness and impairing relaxation [11,12,13]. In type 2 diabetes, higher HbA1c is associated with subclinical systolic dysfunction, as reflected by worse left-ventricular global longitudinal strain (GLS) [14]. In contrast, in predominantly non-diabetic populations, it coexists with diastolic dysfunction and left atrial (LA) enlargement with preserved ejection fraction, interpreted as early myocardial aging [15]. In addition, left-atrial stiffness indices are associated with metabolic and inflammatory markers [16]. Consequently, the use of HbA1c for CV risk assessment is increasingly considered even in individuals without diabetes, although current evidence is inconclusive and requires further study [17].

Consistent data are still lacking for adults without diagnosed diabetes or CVD whose HbA1c lies within the normal range and whose cardiac assessment relies on standard TTE. It is also unclear to what extent any relationships between HbA1c and the echocardiographic phenotype are independent of age, blood pressure, obesity, and other coexisting risk factors that strongly determine chamber remodeling.

The purpose of this study was to assess the relationship between HbA1c and selected echocardiographic parameters in patients without disorders of carbohydrate metabolism. Such aspects have been analyzed, including left ventricular (LV) dimensions and mass, LA parameters, indices of LV systolic and diastolic function, and right ventricular (RV) systolic function. We hypothesized that higher HbA1c, even within the normal range, correlates with a less favorable echocardiographic phenotype, and that the independence of these associations weakens after adjustment for key confounders.

## 2. Materials and Methods

### 2.1. Study Population

The study population included patients hospitalized in the Department of Internal Medicine, Angiology, and Physical Medicine of the Medical University of Silesia in Katowice (Poland) between June 2022 and May 2024.

Inclusion criteria for the study: (1) age at least 18 years; (2) hospitalization for planned internal medicine diagnostics; (3) TTE examination performed during hospitalization; and (4) HbA1c test performed during hospitalization. Exclusion criteria: (1) diagnosed diabetes or prediabetes (based on interview data, medication use, and current fasting venous plasma glucose and HbA1c values); (2) any acute illness or exacerbation of chronic disease within the month preceding their admission to the hospital; (3) tachyarrhythmia; (4) features of decompensated heart failure; (5) respiratory failure; (6) anemia; (7) infection; (8) diagnosed malignant neoplasm; and (9) chronic kidney disease.

### 2.2. Laboratory Tests

The laboratory tests were conducted in the Laboratory of the Specialist Hospital No. 2 in Bytom. Blood samples for these tests were collected in the morning, after fasting for approximately 12 to 14 hours since the last meal.

HbA1c was determined by turbidimetric inhibition immunoassay using the Tina-quant Hemoglobin A1c Gen.3 kit (Roche Diagnostics GmbH, Mannheim, Germany).

### 2.3. Echocardiography

TTE was performed by a cardiologist experienced in this procedure with a Vivid E9 device (GE HealthCare Ultrasound, Chicago, IL, USA) using an M5S-D transducer. Measurements were taken after obtaining standardized views, in accordance with current recommendations [18]. Table 1 summarizes the parameters measured or calculated and used in the final analysis of the current study.

### 2.4. Doppler Ultrasound

An arterial Doppler ultrasound was performed using an RS80 EVO device (Samsung Medison Co., Ltd., Seoul, Republic of Korea) with either a linear probe LA4-18B for carotid arteries or a linear probe LA3-12A for lower extremity arteries. The Doppler ultrasonography of both the carotid and lower limb arteries was conducted following widely accepted guidelines [19,20,21].

### 2.5. Statistical Analysis

Qualitative variables were presented in the form of counts and percentages of individual variants of a given variable. Quantitative variables were presented using the mean and standard deviation (when the distribution did not differ significantly from the normal distribution) or using the median and the first and third quartile values (when the distribution differed significantly from the normal distribution). The conformity of the empirical distribution to the normal distribution was tested using the Shapiro–Wilk test, the Kolmogorov–Smirnov test, and visual analysis of histograms. The Spearman rank correlation test was used to examine the correlation between quantitative variables. *p* < 0.05 was considered statistically significant.

Based on the univariate correlation analysis, a multivariate model was built to better understand the relationship between HbA1c and selected echocardiographic parameters, while accounting for confounding factors. The model was adjusted for age, sex, body mass index (BMI), low-density lipoprotein cholesterol (LDL-C), systolic blood pressure (SBP), hypertension, and smoking, which were selected based on correlation analyses as significant variables. Variables that were not normally distributed were log-transformed.

Statistical analyses were performed using TIBCO Software Inc., Palo Alto, CA, USA (2017), Statistica (data analysis software system), version 13.

## 3. Results

### 3.1. Basic Characteristics of the Study Population

The final analysis included 59 individuals with a mean age of 54.82 years, the majority of whom were women (72.88%). In 31 patients (52.54%), atherosclerosis was diagnosed, defined as the presence of atherosclerotic plaque in any vascular bed (carotid arteries, abdominal aorta, arteries of the lower extremities), as proven by medical imaging, whereas only some patients with documented atherosclerotic plaques were diagnosed in the past with atherosclerotic CVD (four patients reported a history of coronary artery disease). Importantly, no one in the study population had a history of a CV event or invasive revascularization of any vascular bed. Doppler ultrasonography of the carotid arteries was performed in all study participants, while ultrasonography of the abdominal aorta and lower limb arteries was performed in 56 people (94.92%). Detailed data on the study population’s characteristics were collected and are presented in Table 2.

### 3.2. Results of Echocardiography

In the majority of individuals in the study population, TTE did not reveal any significant abnormalities. Only in two patients (3.39%) was the LVEF value below 50%. Similarly, two individuals (3.39%) had signs of RV systolic dysfunction [defined as tricuspid annular plane systolic excursion (TAPSE) < 17 mm]; however, two individuals had missing data regarding TAPSE values. Eight patients (13.56%) had elevated LAVI values (>34 mL/min/m^2^). In 14 people (23.73%), the E/A value was lower than 0.8. The E/A value was greater than 2.0 in only one person (1.69%). No one in the study population had significant valvular defects, pathological pericardial effusion, or additional masses inside the heart chambers. Descriptive statistics for the results of individual echocardiographic parameters in the study population are presented in Table 3.

As presented in Table 3, some cases had missing data. In six cases (10.17%), LV SV values were not recorded in the medical records. In one case (1.69%), LAV and LAVI values were not recorded in the medical records. In two cases (3.39%), TAPSE values were not recorded. In each case, tricuspid regurgitation was assessed; however, in 14 patients (23.72%), regurgitation was not detected, which prevented a reliable measurement of TRV_max_. Parameters related to the mitral inflow profile were measured in all patients; however, in 2 patients (3.39%), the A wave was absent due to atrial fibrillation during the study, so the E/A ratio could not be calculated.

### 3.3. Correlation Between Glycated Hemoglobin and Echocardiographic Parameters

In the study population, a statistically significant positive correlation was found between HbA1c and parameters related to the size of the left ventricle (LV EDD, LV ESD), left ventricular myocardial mass (LVM, LVMI), and the size of the left atrium (LA, LAV, and LAVI).

A significant correlation was also found between HbA1c and parameters related to the mitral inflow profile, in particular, a statistically significant negative correlation between HbA1c and E/A. A significant positive correlation was also found between HbA1c and the mean E/E′ value.

A statistically significant negative correlation was found between HbA1c and right ventricular systolic function (TAPSE), but no significant correlation was found between HbA1c and the parameter related to RV size (RV D1).

The complete results of the correlation analysis between HbA1c value and echocardiographic parameter values are presented in Table 4.

**Figure 1:**
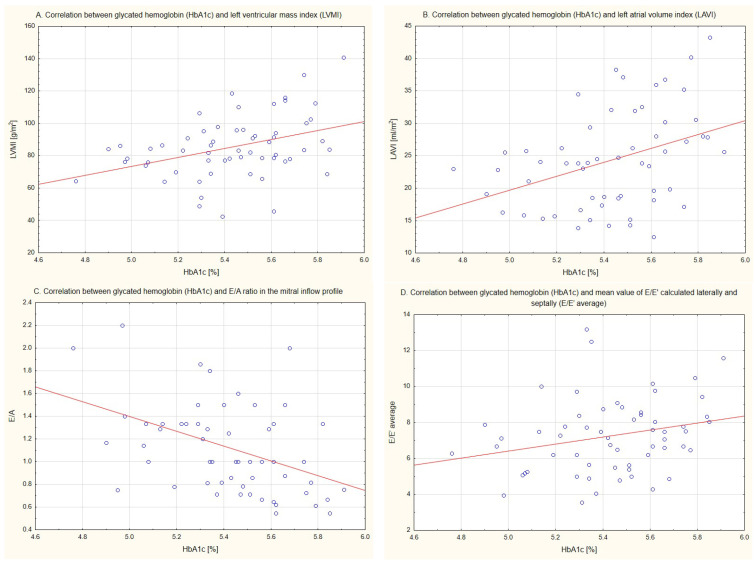
Correlations between glycated hemoglobin (HbA1c) value and selected echocardiographic parameters.

Selected correlations between HbA1c value and some echocardiographic parameters are presented in Figure 1.

### 3.4. Multivariate Analysis

According to the results of multivariate analysis, the created model was found to be significant for the following echocardiographic parameters: LV EDD, LV ESD, LVM, LVMI, LA, log (LAV), log (E/A), E′ septal, log (E/E′ septal), and average E/E′. Full results are presented in Table 5.

For variables for which the model was significant, the influence of HbA1c values on the results was examined. A significant influence of HbA1c on the results was not confirmed, indicating that confounding variables can fully explain the correlation between HbA1c and echocardiographic parameters. The full results are presented in Table 6.

## 4. Discussion

This study examined the association between HbA1c levels and cardiac structure and function, as assessed by TTE, in adults without diabetes. Although mean HbA1c values were within the normal range, there were significant positive correlations between HbA1c and left-ventricular dimensions and mass (LV EDD, LV ESD, LVM, and LVMI), as well as LA parameters (LA, LAV, and LAVI). We also observed significant relationships between HbA1c and diastolic function parameters: negative correlations with E/A and E′, and a positive correlation with E/E′. Interpretation of the correlation between HbA1c and E/A is difficult because both too low (<0.8) and too high (>2.0) E/A values are abnormal and indicate varying degrees of diastolic dysfunction, which may also occur with a normal E/A ratio. Moreover, HbA1c was found to negatively correlate with RV systolic function assessed by tricuspid annular plane systolic excursion (TAPSE). However, these associations were significant only in univariate analysis. The multivariate analysis indicated that confounding factors could explain these associations.

A brief description of the study population requires additional commentary. These were individuals admitted to the Clinic for planned internal medicine diagnostics, without any signs of acute illness. Given the Clinic’s activity profile, these were primarily planned endoscopic gastrointestinal diagnostics or planned non-invasive CV diagnostics. In cases of coexisting CV risk factors or family history, the diagnostic workup was expanded to include CV assessment, even if this assessment was not the primary purpose of hospitalization. Considering the inclusion and exclusion criteria, the analysis included individuals in relatively good health and without a diagnosed severe organic disease. In summary, participants underwent a detailed internal medicine assessment during planned hospitalization, thereby increasing the value of the collected data despite the relatively small sample size.

In this context, HbA1c serves as a marker of metabolic burden and coexisting risk factors. Dubowitz et al. conducted a study in adults without diagnosed diabetes showing that with age, there is an increase in both glucose intolerance and HbA1c levels, independent of other clinical factors such as race, BMI, waist circumference, and lipid profile [10]. Myocardial fibrosis is a key pathophysiological mechanism underlying structural and functional cardiac abnormalities in type 2 diabetes. Excess metabolism and elevated glucose residues act synergistically to promote the formation of AGEs. AGEs can crosslink extracellular-matrix proteins and impair their degradation by matrix metalloproteinases, increasing myocardial stiffness and promoting early diastolic dysfunction [22,23]. Li et al. showed that poor glycemic control correlates with diffuse myocardial fibrosis and load-related myocardial injury [24]. An increase in LVM raises myocardial oxygen demand and thereby CV risk. In women with prediabetes, echocardiographic signs of increased LVM seen in men with the same condition have not been demonstrated, suggesting that more pronounced glucose dysregulation is required for LV hypertrophy to develop [25].

In multivariable analysis, although the model was significant for many structural and functional parameters (including LVMI, LA, log LAV, log E/A, septal E′, and average E/E′), the effect of HbA1c was no longer independent after adjustment for covariates. It suggests that the observed correlations may partly derive from other factors such as age, blood pressure, and body mass, which strongly influence chamber remodeling. Population data indicate that these factors, together with aging and subclinical inflammation, are the main determinants of myocardial structure and function [6].

In type 2 diabetes populations, an independent association between HbA1c and subclinical LV systolic dysfunction has been repeatedly shown, particularly for GLS. Gao et al. similarly reported that HbA1c is independently associated with worse LV GLS, even after adjustment for LA parameters, confirming the link between glycemia and subclinical myocardial injury [14]. Relationships between chronic glycemia and cardiac aging have also been described in predominantly non-diabetic cohorts: higher HbA1c coexists with subtle diastolic dysfunction and LA enlargement with preserved LV ejection fraction, interpreted as early myocardial aging [15]. Our results align with this pattern, especially for E/A, E′, and LAVI. Variability of GLS findings in prediabetes highlights the influence of metabolic disturbances such as obesity and insulin resistance [26]. In our cohort, where GLS was not routinely assessed, significant associations involved mainly LV and LA dimensions and diastolic indices, consistent with the roles of age, blood pressure, and obesity as dominant determinants of chamber remodeling. Population data indicate that age and elevated blood pressure per se shape chamber size and diastolic function, thereby attenuating the impact of glycemia in multivariable models [6]. For the LA, stiffness and phasic function matter in addition to volume. Studies of the LA stiffness index have shown metabolic and inflammatory associations in hypertension and diabetes, including inflammatory responses [16], which may explain our positive correlations between HbA1c and LAV/LAVI.

HbA1c and cardiac aging may be linked through the accumulation of AGEs. Like HbA1c, AGEs arise from non-enzymatic glycation. They form gradually and accumulate in vessel walls; their deposition can increase arterial stiffness, limit myocardial perfusion, and ultimately lead to cardiac dysfunction [12,13]. In our cohort, more than half of the participants had imaging evidence of atherosclerosis despite no symptomatic CVD, suggesting that subclinical metabolic and structural changes may co-occur as an early stage of a cardiometabolic phenotype. In non-diabetic populations, markers of intensified glycation better reflect CV risk: a high hemoglobin glycation index (HGI) correlates with worse LV function [15]. Hyperglycemia and AGEs amplify oxidative stress, inflammation, and extracellular matrix fibrosis, increasing myocardial and atrial stiffness and impairing relaxation [27]. The link between glycation and cardiac remodeling is also supported by data showing that AGE levels correlate with the severity of aortic stenosis (AS), connecting glycation with valvular disease progression and echocardiographic parameters [28]. Growing evidence indicates that elevated HbA1c, even at early dysglycemia, is associated with aortic-valve calcification in the general population, mainly when dysglycemia is classified by HbA1c values [29]. In patients with severe AS assessed by computed tomography, relationships among glycemia, HbA1c, and AGEs with calcification phenotype and echocardiographic indices of AS severity suggest potential interactions between advanced glycation and the valvular phenotype [28]. Chronic hyperglycemia and non-enzymatic glycation promote oxidative stress, fibrosis, and endothelial dysfunction, leading to myocardial remodeling and impaired ventricular filling [11]. Although our study relied on standard TTE, cardiac magnetic resonance imaging data in hypertrophic cardiomyopathy show progressive increases in fibrosis (native T1 and extracellular volume fraction) and worsening LV strain across normoglycemia, prediabetes, and diabetes. The extracellular volume fraction correlated positively with HbA1c. These changes are subtle and often invisible on conventional late gadolinium enhancement imaging, indicating early diffuse matrix remodeling [27]. The absence of independent associations between HbA1c and echocardiographic parameters in our model may therefore reflect the limited sensitivity of standard TTE to detect subclinical tissue abnormalities.

We observed a negative correlation between HbA1c and TAPSE in univariable analysis; however, this relationship did not persist after adjustment. Recent studies in type 2 diabetes using two-dimensional speckle-tracking echocardiography have confirmed that higher HbA1c is independently associated with impaired RV free-wall longitudinal strain, and RV dysfunction is significantly more frequent at HbA1c ≥ 7% [30]. In prediabetes, RV strain parameters can detect dysfunction earlier than traditional indices such as TAPSE, which may still be normal [31]. It explains why, in our cohort, based mainly on conventional measurements, the association with HbA1c did not remain independent after adjustment—the lack of RV strain assessment likely limited sensitivity to subtle changes. An association between HbA1c levels and diastolic function is seen in older adults even before overt CVD. Aging of the CV system itself increases the risk of future CV conditions [6,32].

From a thromboembolic risk perspective, higher HbA1c levels in atrial fibrillation are associated with reduced left atrial appendage flow velocity and potentially impaired contractility, further supporting the link between glycemia and atrial remodeling [33]. Zhang et al., in a study involving patients with hypertension—both with and without diabetes—found that higher HbA1c levels were independently associated with LA dysfunction. These findings are consistent with our results and further emphasize the clinical value of routine HbA1c measurement in assessing CV risk, even among individuals without diagnosed diabetes [34].

### Limitations of This Study

The study we conducted has significant limitations. First of all, a significant limitation of the study is its relatively small sample size, which precludes further subgroup analyses. Our cohort represented a relatively low-risk group without clinically overt CVD or advanced renal disease, with a high proportion of normal LV EF and LAVI values and a relatively young mean age. The relatively healthy, homogeneous study group makes it difficult to detect subtle abnormalities in the morphology and function of cardiac structures. Such differences could be better identified by employing more sensitive echocardiographic techniques, such as speckle-tracking echocardiography, or by combining imaging modalities. The lack of such techniques also remains a significant limitation of our study. In particular, basing the assessment of RV systolic function solely on TAPSE without using RV strain, as well as the lack of assessment of various aspects of LA function (reservoir, conduit, booster) using LA strain, constitute significant limitations. Additionally, covariates such as age, systolic blood pressure, BMI, and lipid profile strongly influence LAVI, E/E′, and LV dimensions, which may explain the correlations with HbA1c observed in univariate analysis. It is necessary to conduct studies on a larger patient population to identify subgroups based on the constellation of coexisting cardiovascular risk factors. Finally, HbA1c does not fully capture the heterogeneity of glycation; the “high-glycation” phenotype observed in some individuals may be better identified by the hemoglobin glycation index (HGI), which has been linked to energy metabolism and vascular stiffness [5,29].

## 5. Conclusions

In summary, our findings indicate that even small differences in HbA1c within the normal range may correlate with structural and diastolic cardiac parameters. After adjustment for confounders, however, this relationship loses independence, suggesting that normoglycemic HbA1c reflects overall metabolic burden rather than a direct driver of cardiac remodeling. Further studies using more sensitive imaging methods, such as two-dimensional speckle-tracking echocardiography or cardiac magnetic resonance imaging with T1 and extracellular volume fraction mapping, are needed to confirm these observations and elucidate early mechanisms linking glycation with myocardial remodeling.

## Figures and Tables

**Table 1:** List of parameters used to assess individual aspects of the morphology and function of heart structures.

Assessment Aspect	Parameters
Left ventricular size and geometry	Left ventricular end diastolic diameter (LV EDD)
	Left ventricular end systolic diameter (LV ESD)
	Interventricular septum thickness (IVS)
	Posterior wall thickness (PW)
	Relative wall thickness (RWT)
Left ventricular mass	Left ventricular mass (LVM)
	Left ventricular mass index (LVMI)
Left ventricular systolic function	Left ventricular stroke volume (LV SV)
	Left ventricular ejection fraction (LV EF)
	Left ventricular fractional shortening (LV FS)
	Lateral S′ velocity detected by tissue Doppler echocardiography (S′ lateral)
	Septal S′ velocity detected by tissue Doppler echocardiography (S′ septal)
Left ventricular diastolic function	Mitral inflow E/A ratio (E/A)
	Mean value of E/E′ calculated laterally and septally (E/E′ average)
	Peak tricuspid regurgitation velocity (TRV_max_)
Left atrial size	Left atrial dimension (LA)
	Left atrial volume (LAV)
	Left atrial volume index (LAVI)
Right ventricular size	Basal right ventricular diameter (RV D1)
Right ventricular function	Tricuspid annular plane systolic excursion (TAPSE)

**Table 2:** Demographic and clinical characteristics of the study population.

Variable		N (%)/Mean (SD)/ Median (Q1; Q3)
Age [years]		54.82 (17.34)
Gender		Females: 43 (72.88%) Males: 16 (27.12%)
Arterial hypertension		30 (50.85%)
Smoking	Current smoker	15 (25.86%)
	Former smoker	18 (31.03%)
	Never	25 (43.1%)
Atherosclerosis		31 (52.54%)
Atrial fibrillation	Paroxysmal	2 (3.39%)
	Persistent	2 (3.39%)
Body mass index (BMI) [kg/m^2^]		25.81 (4.38)
Waist-to-hip ratio (WHR)		0.89 (0.091)
Systolic blood pressure [mmHg]		128.0 (115.0; 142.0)
Diastolic blood pressure [mmHg]		77.0 (72.0; 83.0)
Total cholesterol (TC) [mg/dL]		195.29 (37.64)
Low-density lipoprotein cholesterol (LDL-C) [mg/dL]		124.72 (34.57)
High-density lipoprotein cholesterol (HDL-C) [mg/dL]		59.65 (14.62)
Triglyceride (TG) [mg/dL]		100.0 (70.0; 134.0)
Non-HDL-C [mg/dL]		136.9 (110.8; 160.0)
Uric acid [mg/dL]		4.8 (3.4; 5.8)
Glycated hemoglobin [%]		5.46 (5.29; 5.62)

**Table 3:** Echocardiographic results in the study population.

Parameter	N	Mean (SD)/Median (Q1; Q3)	Minimal Value	Maximal Value
IVS [mm]	59	10.0 (10.0; 10.0)	6.0	12.0
PW [mm]	59	10.0 (10.0; 10.0)	6.0	12.0
LV EDD [mm]	59	45.61 (5.53)	34.0	61.0
LV ESD [mm]	59	34.03 (5.19)	25.0	47.0
LVM [g]	59	154.0 (39.2)	63.74	263.65
LVMI [g/m^2^]	59	85.38 (19.08)	42.58	140.8
RWT	59	0.433 (0.071)	0.262	0.6
LV SV [mL]	53	72.82 (17.02)	38.0	112.12
LV EF [%]	59	56.0 (54.0; 60.0)	40.0	62.0
LV FS [%]	59	26.09 (19.61; 30.43)	9.62	36.17
LA [mm]	59	31.86 (4.066)	24.0	42.0
LAV [ml]	58	43.54 (34.29; 50.94)	22.04	84.51
LAVI [mL/m^2^]	58	23.91 (18.46; 28.0)	12.52	43.24
RV D1 [mm]	59	30.19 (3.86)	22.0	41.0
TAPSE [mm]	57	24.0 (23.0; 24.0)	10.0	26.0
TRV_max_ [m/s]	45	2.1 (1.8; 2.4)	1.4	2.9
E [m/s]	59	0.8 (0.6; 0.9)	0.44	1.3
A [m/s]	57	0.7 (0.6; 0.8)	0.4	1.1
E/A	57	1.0 (0.78; 1.33)	0.55	2.2
S′ septal [cm/s]	59	9.0 (8.0; 10.0)	6.0	16.0
S′ lateral [cm/s]	59	10.0 (8.0; 12.0)	6.0	17.0
E′ septal [cm/s]	59	10.0 (8.0; 13.0)	3.0	18.0
E′ lateral [cm/s]	59	11.0 (9.0; 15.0)	5.0	20.0
E/E′ septal	59	7.5 (6.0; 8.89)	3.75	14.75
E/E′ lateral	59	6.43 (5.0; 8.18)	3.33	13.2
E/E′ average	59	7.26 (2.061)	3.54	13.2

Abbreviations: IVS—interventricular septum; PW—posterior wall thickness; LV EDD—left ventricular end-diastolic diameter; LV ESD—left ventricular end-systolic diameter; LVM—left ventricular mass; LVMI—left ventricular mass index; RWT—relative wall thickness; LV SV—left ventricular stroke volume; LV EF—left ventricular ejection fraction; LV FS—left ventricular fractional shortening; LA—left atrial dimension; LAV—left atrial volume; LAVI—left atrial volume index; RV D1—basal right ventricular diameter; TAPSE—tricuspid annular plane systolic excursion; TRV_max_—peak tricuspid regurgitation velocity; E—mitral E-wave velocity; A—mitral A-wave velocity; S′ septal—septal S′ velocity (tissue Doppler echocardiography); S′ lateral—lateral S′ velocity (tissue Doppler echocardiography); E′ septal—early diastolic mitral annular velocity measured at the septal wall of the heart; E′ lateral—early diastolic mitral annular velocity measured at the lateral wall of the heart; E/E′ average—mean value of E/E′ calculated laterally and septally.

**Table 4:** Correlations between glycated hemoglobin percentage and selected echocardiographic parameters.

Parameter	N	R	*p*
IVS	59	0.091	0.495
PW	59	0.037	0.282
LV EDD	59	0.348	0.007
LV ESD	59	0.342	0.008
LVM	59	0.303	0.02
LVMI	59	0.378	0.003
RWT	59	−0.226	0.085
LV SV	53	−0.036	0.8
LV EF	59	−0.24	0.067
LV FS	59	−0.084	0.53
LA	59	0.344	0.008
LAV	58	0.369	0.004
LAVI	58	0.401	0.002
RV D1	59	0.068	0.611
TAPSE	57	−0.417	0.001
TRV_max_	45	0.198	0.192
E	59	−0.286	0.028
A	57	0.384	0.003
E/A	57	−0.427	0.001
S′ septal	59	−0.2	0.135
S′ lateral	59	−0.22	0.093
E′ septal	59	−0.389	0.002
E′ lateral	59	−0.367	0.004
E/E′ septal	59	0.263	0.044
E/E′ lateral	59	0.19	0.151
E/E′ average	59	0.262	0.045

Abbreviations: IVS—interventricular septum thickness; PW—posterior wall thickness; LV EDD—left ventricular end-diastolic diameter; LV ESD—left ventricular end-systolic diameter; LVM—left ventricular mass; LVMI—left ventricular mass index; RWT—relative wall thickness; LV SV—left ventricular stroke volume; LV EF—left ventricular ejection fraction; LV FS—left ventricular fractional shortening; LA—left atrial dimension; LAV—left atrial volume; LAVI—left atrial volume index; RV D1—basal right ventricular diameter; TAPSE—tricuspid annular plane systolic excursion; TRV_max_—peak tricuspid regurgitation velocity; E—mitral inflow E-wave velocity; A—mitral inflow A-wave velocity; S′ septal—septal S′ velocity (tissue Doppler echocardiography); S′ lateral—lateral S′ velocity (tissue Doppler echocardiography); E′ septal—early diastolic mitral annular velocity measured at the septal wall of the heart; E′ lateral—early diastolic mitral annular velocity measured at the lateral wall of the heart; E/E′ average—mean value of E/E′ calculated laterally and septally.

**Table 5:** Results of the multivariate analysis model significance test for explaining individual echocardiographic parameters. The model was adjusted for age, sex, body mass index (BMI), low-density lipoprotein cholesterol serum concentration (LDL-C), systolic blood pressure (SBP), hypertension, and smoking.

Parameter	Multiple R^2^	*p*
LV EDD	0.357	0.01
LV ESD	0.421	0.001
LVM	0.455	<0.001
LVMI	0.356	0.007
RWT	0.177	0.35
LV SV	0.173	0.47
LV EF	0.260	0.08
LV FS	0.258	0.08
LA	0.531	<0.001
log (LAV)	0.325	0.02
LAVI	0.274	0.065
log (E/A)	0.519	<0.001
log (S′ septal)	0.225	0.159
log (S′ lateral)	0.243	0.112
E′ septal	0.548	<0.001
log (E/E′ septal)	0.315	0.022
log (E/E′ lateral)	0.264	0.072
E/E′ average	0.303	0.03

Abbreviations: LV EDD—left ventricular end-diastolic diameter; LV ESD—left ventricular end-systolic diameter; LVM—left ventricular mass; LVMI—left ventricular mass index; RWT—relative wall thickness; LV SV—left ventricular stroke volume; LV EF—left ventricular ejection fraction; LV FS—left ventricular fractional shortening; LA—left atrial dimension; LAV—left atrial volume; LAVI—left atrial volume index; E—mitral inflow E-wave velocity; A—mitral inflow A-wave velocity; S′ septal—septal S′ velocity (tissue Doppler echocardiography); S′ lateral—lateral S′ velocity (tissue Doppler echocardiography); E′ septal—early diastolic mitral annular velocity measured at the septal wall of the heart; E′ lateral—early diastolic mitral annular velocity measured at the lateral wall of the heart; E/E′ average—mean value of E/E′ calculated laterally and septally.

**Table 6:** Results of the multivariate analysis model significance test for explaining individual echocardiographic parameters.

Parameter	β	95% CI	*p*
LV EDD	0.12	−0.17; 0.41	0.402
LV ESD	0.15	−0.12; 0.43	0.27
LVM	0.09	−0.18; 0.36	0.503
LVMI	0.13	−0.16; 0.42	0.379
LA	0.01	−0.24; 0.26	0.92
log (LAV)	0.127	−0.174; 0.427	0.4
log (E/A)	−0.09	−0.34; 0.17	0.49
E′ septal	0.02	−0.23; 0.26	0.887
log (E/E′ septal)	−0.004	−0.304; 0.295	0.976
E/E′ average	−0.06	−0.36; 0.25	0.714

Abbreviations: LV EDD—left ventricular end-diastolic diameter; LV ESD—left ventricular end-systolic diameter; LVM—left ventricular mass; LVMI—left ventricular mass index; LA—left atrial dimension; LAV—left atrial volume; E—mitral inflow E-wave velocity; A—mitral inflow A-wave velocity; E′ septal—early diastolic mitral annular velocity measured at the septal wall of the heart; E/E′ average—mean value of E/E′ calculated laterally and septally.

## Data Availability

The data presented in this study are available upon request from the corresponding author.

## Abbreviations

The following abbreviations are used in this manuscript:

A	Mitral inflow A-wave velocity
AGE	Advanced glycation end product
CV	Cardiovascular
CVD	Cardiovascular disease
E′ lateral	Early diastolic mitral annular velocity measured at the lateral wall of the heart
E′ septal	Early diastolic mitral annular velocity measured at the septal wall of the heart
E/E′ average	Mean value of E/E′ calculated laterally and septally
E	Mitral inflow E-wave velocity
GLS	global longitudinal strain
HbA1c	Glycated hemoglobin percentage
HGI	Hemoglobin glycation index
IVS	Interventricular septum thickness
LA	Left atrial dimension
LAV	Left atrial volume
LAVI	Left atrial volume index
LV	Left ventricular
LV EDD	Left ventricular end-diastolic diameter
LV EF	Left ventricular ejection fraction
LV ESD	Left ventricular end-systolic diameter
LV FS	Left ventricular fractional shortening
LV SV	Left ventricular stroke volume
LVM	Left ventricular mass
LVMI	Left ventricular mass index
PW	Posterior wall thickness
Q1	The first quartile
Q3	The third quartile
RV	Right ventricular
RV D1	Basal right ventricular diameter
RWT	Relative wall thickness
S′ lateral	Lateral S′ velocity (tissue Doppler echocardiography)
S′ septal	Septal S′ velocity (tissue Doppler echocardiography)
SD	Standard deviation
TAPSE	Tricuspid annular plane systolic excursion
TRV_max_	Peak tricuspid regurgitation velocity
TTE	Transthoracic echocardiography
